# Vaccination with an HIV T-Cell Immunogen (HTI) Using DNA Primes Followed by a ChAdOx1-MVA Boost Is Immunogenic in Gut Microbiota-Depleted Mice despite Low IL-22 Serum Levels

**DOI:** 10.3390/vaccines11111663

**Published:** 2023-10-30

**Authors:** Aleix Elizalde-Torrent, Alessandra Borgognone, Maria Casadellà, Luis Romero-Martin, Tuixent Escribà, Mariona Parera, Yaiza Rosales-Salgado, Jorge Díaz-Pedroza, Francesc Català-Moll, Marc Noguera-Julian, Christian Brander, Roger Paredes, Alex Olvera

**Affiliations:** 1 Irsicaixa—AIDS Research Institute, 08916 Barcelona, Spain; aelizalde@irsicaixa.es (A.E.-T.); aborgognone@irsicaixa.es (A.B.); mcasadella@irsicaixa.es (M.C.); luisrommar@gmail.com (L.R.-M.); tescriba@irsicaixa.es (T.E.); mparera@irsicaixa.es (M.P.); fcatala@irsicaixa.es (F.C.-M.); mnoguera@irsicaixa.es (M.N.-J.); cbrander@irsicaixa.es (C.B.); rparedes@irsicaixa.es (R.P.); 2 Departament de Biologia Cellular, Fisiologia i Immunologia, Universitat Autonoma de Barcelona (UAB), 08193 Cerdanyola del Valles, Spain; 3 Fundació Institut d’Investigació en Ciències de la Salut Germans Trias i Pujol (IGTP), 08916 Badalona, Spain; yrosales@igtp.cat (Y.R.-S.); jdiaz@igtp.cat (J.D.-P.); 4 Facultat de Medicina, Universitat de Vic—Universitat Central de Catalunya (UVic-UCC), 08500 Vic, Spain; 5 CIBERINFEC—ISCIII, 28029 Madrid, Spain; 6 Institució Catalana de Recerca i Estudis Avançats (ICREA), 08010 Barcelona, Spain; 7 Aelix Therapeutics, 08028 Barcelona, Spain; 8 Center for Global Health and Diseases, Department of Pathology, Case Western Reserve University, Cleveland, OH 44106, USA; 9 Fight AIDS Foundation, Infectious Diseases Department, Germans Trias i Pujol University Hospital, 08916 Badalona, Spain; 10 Department of Infectious Diseases Service, Germans Trias i Pujol University Hospital, 08916 Badalona, Spain; 11 Facultat de Ciències, Tecnologia i Enginyeries, Universitat de Vic—Universitat Central de Catalunya (UVic-UCC), 08500 Vic, Spain

**Keywords:** microbiota, HIV, vaccine, T-cell, IFNγ, IL-22, *Roseburia*

## Abstract

Despite the important role of gut microbiota in the maturation of the immune system, little is known about its impact on the development of T-cell responses to vaccination. Here, we immunized C57BL/6 mice with a prime-boost regimen using DNA plasmid, the Chimpanzee Adenovirus, and the modified Vaccinia Ankara virus expressing a candidate HIV T-cell immunogen and compared the T-cell responses between individuals with an intact or antibiotic-depleted microbiota. Overall, the depletion of the gut microbiota did not result in significant differences in the magnitude or breadth of the immunogen-specific IFNγ T-cell response after vaccination. However, we observed marked changes in the serum levels of four cytokines after vaccinating microbiota-depleted animals, particularly a significant reduction in IL-22 levels. Interestingly, the level of IL-22 in serum correlated with the abundance of *Roseburia* in the large intestine of mice in the mock and vaccinated groups with intact microbiota. This short-chain fatty acid (SCFA)-producing bacterium was significantly reduced in the vaccinated, microbiota-depleted group. Therefore, our results indicate that, although microbiota depletion reduces serum levels of IL-22, the powerful vaccine regime used could have overcome the impact of microbiota depletion on IFNγ-producing T-cell responses.

## 1. Introduction

Vaccines are one of the most successful tools to prevent infectious diseases, but their capacity to induce strong, long-lasting, antibody and cytotoxic T-cell-mediated immune responses can vary between individuals. Vaccine immunogenicity and efficacy are influenced by many factors, including host genetics and cross-reactivity with non-targeted microbes. Growing evidence also indicates that the composition of the gut microbiota can also influence responses to vaccination [1,2].

It is well known that the gut microbiota has a crucial impact on the function of the immune system [3,4,5,6,7], promoting the development of intestinal lymphoid tissues as well as the maturation of myeloid and lymphoid cells [8,9,10,11,12]. Furthermore, gut microbes persistently stimulate antigen-presenting cells (APC), CD4+ T, and B cells, thereby shaping effective humoral responses [3,13]. Accordingly, intestinal dysbiosis has been related to an increasing number of diseases, including autoimmunity, chronic inflammation disorders, allergies, and asthma [7,14,15,16,17,18,19,20,21,22]. However, the exact mechanisms by which intestinal bacteria can influence immune responses to other microorganisms, tumors, or vaccines remain poorly understood.

Growing evidence also shows that the gut microbiome plays a crucial role in maintaining the epithelial barrier and the regulation of innate immunity [23,24,25,26,27,28] by reducing neutrophil inflammatory responses and modulating macrophage, dendritic cells (DC), and the natural killer (NK) cell function [9,10,29,30,31]. Accordingly, the microbiota is thought to prevent potentially harmful innate immune responses and inflammation that are triggered in response to pathogenic bacteria [32,33]. Interestingly, the gut microbial community also has the capacity to influence adaptive immunity [1,11,12,13]. For instance, the recognition of microbiota-derived pathogen-associated molecular patterns (PAMP) can help generate antibody responses and mediate isotype class switching and plasma cell differentiation after vaccination [1,34]. Furthermore, different bacterial products can regulate the production of specific cytokines and drive the differentiation of specific T-cell subtypes, especially Th17 and Treg cells [2,35]. In addition to PAMP, other microbiota-derived metabolites such as short-chain fatty acids (SCFA), especially butyrate and propionate, have been shown to exert immunomodulatory properties on macrophages, dendritic cells, innate lymphoid cells, T- and B-cells [36,37,38,39].

In line with this, several reports have described the influence of the gut microbiome on the immunogenicity of SARS-CoV-2, Rotavirus, Human Papillomavirus, Polio, Tetanus, Pneumococcus and *Vibrio cholera* vaccines [40,41,42,43,44,45,46,47,48]. Moreover, microbiota dysbiosis caused by antibiotic use has been shown to have detrimental effects on vaccine response [2] and to reduce antibody levels and CD8+ T-cell responses to influenza vaccination [35,49]. There are also some studies in humans linking specific bacteria genera, such as *Streptococcus*, *Actinobacteria*, and *Roseburia*, to changes in vaccine response [2,50,51,52,53]. In the case of HIV, the HVTN 505 vaccine trial showed the existence of bacterial epitopes that cross-reacted with HIV, driving immunodominant anti-Gp41 antibody responses after vaccination [54]. Furthermore, in the HVTN 096 clinical trial, vaccine-specific IgG levels were correlated with three clusters of microbial families [55]. Indeed, in an unrelated study, the levels of neutralizing antibodies to a HIV vaccine were correlated with the abundance of *Eubacterium* in the stool and *Prevotella* in the skin [56].

However, comparable evidence linking microbiota composition and vaccine-induced T-cell responses remains weak. One of the few reports in this regard showed that intestinal dysbiosis decreased T-cell responses to BCG vaccination [57]. Furthermore, the gut microbial fructose/rhamnose degradation pathway is inversely correlated with BNT162b2 SARS-CoV-2 vaccine-induced T-cell responses [58]. Moreover, the potentially predictive signature of the Bacteroidales to Clostridiales ratio has been associated with HIV-1 reservoir size and immune-mediated viral control after vaccination with an HIV T-cell vaccine (MVA.HIVconsv) [59]. In addition, responders to a dendritic cell-based therapeutic HIV vaccine showed enriched levels of Bacteroidetes and Verrucomicrobia, whereas non-responders were enriched for Tenericutes and Actinobacteria [60].

The present study was designed to explore potential changes in T-cell responses to vaccination-induced by gut microbiota depletion. For this, the HIVACAT T-cell immunogen (HTI) was used as a model antigen for vaccination in mice. The HTI amino acid sequence was designed as the concatenation of 16 clade B consensus HIV Gag, Pol, Vif, and Nef protein fragments. The selected fragments were preferentially targeted by T-cells from individuals showing the natural control of HIV infection, measured as IFNγ production in peripheral blood mononuclear cells (PBMC) [61,62,63]. The HTI open reading frame (ORF) has been inserted into different vaccine vectors [62], including DNA plasmid, the Chimpanzee Adenovirus, and modified Vaccinia Ankara vaccine vectors. These HTI-expressing vectors have been tested in different prime-boost strategies in mice [62,64,65,66,67,68], non-human primates [62], and humans [69,70], showing strong immunogenicity in all cases.

Thus, herein, we present a proof-of-concept study in which the impact of the gut microbiota on vaccine immunogenicity is evaluated, comparing T-cell responses and cytokine production between mice with intact or antibiotic-depleted microbiota.

## 2. Materials and Methods

### 2.1. Experimental Design

A total of 60 (30 females and 30 males) C57BL/6JOlaHsd, 6-week-old, specific pathogen-free (SPF) mice grown in the same animal facility were purchased at ENVIGO. Animals were housed in the level 3 biological containment animal facility of the Comparative Medicine and Bioimage Centre of Catalunya (CMCiB) under controlled conditions (temperature 22 ± 2 °C, pressure −20 pascals, 12 h light/dark cycles), with access to drinking water and food ad libitum. Upon arrival, they were non-randomly divided into 12 different cages (6 for males and 6 for females), with a maximum of 5 animals per cage.

After 1 week of acclimatization and before vaccination, animals received a mouse-to-mouse fecal microbiota transplant (FMT) to homogenize microbiota among the animals as conducted by Le Roy et al. [71]. Briefly, feces from male and female, non-vaccinated, SPF, donor mice (n = 10) were collected, mixed, aliquoted (350 mg vials), and preserved at −80 °C until use. A reduction in natural murine intestinal bacterial microbiota was performed to facilitate FMT engraftment via the oral gavage administration of four antibiotics (ampicillin, amikacin, metronidazole, and vancomycin, 10 mg each antibiotic/mouse/day) for 5 days. On the day of the transplant, FMT donor feces were thawed and homogenized in 2.3 mL of sterile physiological saline. After centrifugation (500× *g*, 1 min), the supernatant was recovered and administered via oral gavage (100 µL/animal). The bacterial load in FMT was measured using the LIVE/DEAD^®^ BacLight™ Bacterial Viability and Counting Kit (Invitrogen) in an LSR Fortessa SORP (BD) flow cytometer in the Flow Cytometry Service of the Institut d’Investigació Germans Trias i Pujol (IGTP).

**Figure 1:**
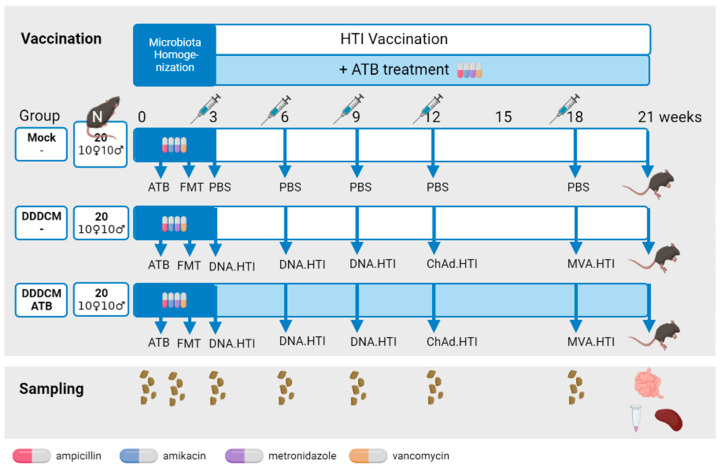
Animal treatments, vaccination, and sampling schedule. All groups received a fecal microbiota transplant (FMT) from the same donor pool prior to vaccination. Afterward, mock animals were vaccinated with PBS while DDDCM and DDDCM-ATB groups received three DNA.HTI primes and ChAdOx1.HTI-MVA.HTI boost regimen. The DDDCM-ATB group also received antibiotic treatment (ATB) in drinking water throughout the vaccination period. Feces were collected before and after FMT as well as before each vaccination. At the last study time point (week 21), the intestinal content, serum, and spleen were collected. Created with BioRender.com.

After FMT, animal cages were divided into three experimental groups (Figure 1), and the immunogenicity of HTI vaccines was assessed. Based on previous results [62,64,65,69], we selected three DNA.HTI primes, followed by a ChAdOx1.HTI and an MVA.HTI boost as the most immunogenic vaccination regime (DDDCM.HTI). Mice in the mock group (n = 20, 10 females and 10 males) were open-label intramuscularly injected with phosphate-buffered saline (PBS) as a control. Those in the DDDCM (n = 20, 10 females and 10 males) and DDDCM-ATB (n = 20, 10 females and 10 males) groups were vaccinated with the following: (i) three DNA.HTI (100 μg/animal) primes, (ii) one boost with ChAdOx1.HTI (1 × 10^9^ VP/animal), and (iii) a second boost with MVA.HTI (1 × 10^6^ pfu/animal), which were all administered intramuscularly, with the dose split equally in both caudal thigh muscles. Mock and DDDCM groups were assayed in two separate batches of 10 animals. All the DDDCM-ATB groups were assayed together with the second batch. DNA.HTI vaccinations were administered in three-week intervals, whereas ChAdOx1.HTI and MVA.HTI administrations were separated by six weeks (Figure 1). The DDDCM-ATB group additionally received a mix of four wide-spectrum antibiotics (ATB: ampicillin and metronidazole: 1 g/L; amikacin and vancomycin: 500 mg/L) ad libitum in drinking water after FMT until the last study time point at week 21 [72]. Animal euthanasia and the sample collection of the intestinal content, blood, and spleen were aseptically performed three weeks after the last immunization. Spleen cells were processed and cryopreserved to be tested in IFNγ ELISPOT assays.

We additionally tested for differences in vaccine response between sexes in six independent validation groups of C57BL/6 mice (ENVIGO). Briefly, these additional experiments included five groups of mice with an unmodified microbiota vaccinated with the following: (i) three DNA.HTI primes separated by three weeks (DDD, n = 5 males and 5 females), (ii) three DNA.HTI primes followed by an MVA.HTI boost separated by three weeks (DDDM, n = 5 males and 5 females), (iii) one ChAdOx1.HTI prime (C, n = 5 males and 4 females), (iv) one ChAdOx1.HTI prime followed by an MVA.HTI boost separated by 6 weeks (CM, n = 4 males and 5 females), (v) three DNA.HTI primes followed after three weeks by a ChAdOx1.HTI-MVA.HTI boost separated by six weeks (DDDCM, n = 4 males and 5 females). A sixth group (vi) was used where the microbiota was depleted using ATB as before and where animals were vaccinated with three DNA.HTI prime vaccinations, followed by ChAdOx1.HTI immunization after six weeks and an MVA.HTI dose after a further three weeks (DDDCM-ATB2, n = 5 males and 5 females). Animal euthanasia and aseptic spleen collection were performed after three weeks. Spleen cells were processed and tested fresh in IFNγ ELISPOT assays.

### 2.2. Sample Collection and Processing

Fecal samples deposited overnight were collected from each cage at animal arrival (week 0) after antibiotic conditioning prior to FMT (week 1) and before every vaccination (weeks 3, 6, 9, 12, and 18). Feces were pooled and stored at −80 °C until use (Figure 1). In addition, intestinal content (caecum large and small intestine samples) was collected at the final study time point. The serum was separated from the rest of the blood via centrifugation (10,000× *g*, 5 min) in Serum Gel S/1.1 tubes (Sarstedt) and stored at −80 °C. The spleen was recovered, and cells were isolated via mechanical disruption and passage through a 40 μm cell strainer (Falcon) using a 5 mL rubber syringe plunger. Following the elimination of red blood cell (RBC) contamination using an RBC lysis buffer (17 mM Tris and 0.14 M NH_4_Cl; Sigma-Aldrich Corp, Kanagawa, Japan), spleen cells were washed twice with R10 (RPMI 1640, supplemented with 10% fetal bovine serum (FBS), 2 mM of L-glutamine, 100 U/mL of penicillin and 100 µg/mL of streptomycin; Gibco, Dublin, Ireland). When needed, spleen cells were cryopreserved in FBS with 10% DMSO (Sigma) and stored in liquid nitrogen until use in IFNγ ELISPOT assays. Caecum from the large and small intestine were collected, and their content was stored separately at −80 °C.

### 2.3. DNA Extraction and 16S rRNA Sequencing

DNA was extracted from the following: (i) longitudinal fecal samples from mock (pooled feces from 1 cage of females and a pool from 1 cage of males for each time point), DDDCM (1 pooled cage of females and 1 pooled cage of males for each time point), and DDDCM-ATB (2 pooled cages of females and 2 pooled cages of males for each time point) groups, as well as from (ii) samples of individual caecum and small and large intestine content at the last study time point from mock (n = 10, 5 females, and 5 males) DDDCM (n = 10, 5 females, and 5 males) and DDDCM-ATB (n = 16, 7 females, and 9 males) groups using the QIAamp DNA Stool Kit (Qiagen, Hilden, Germany). The extracted DNA was prepared for sequencing following the 16S Metagenomic Sequencing Library Preparation protocol [73]. Briefly, the 16S rRNA gene V3–V4 hypervariable region was amplified using specific primers and introducing an Illumina adapter nucleotide overhang: 16S_F 5′-(TCG TCG GCA GCG TCA GAT GTG TAT AAG AGA CAG CCT ACG GGN GGC WGC AG)-3′ and 16S_R 5′-(GTC TCG TGG GCT CGG AGA TGT GTA TAA GAG ACA GGA CTA CHV GGG TAT CTA ATC C)-3′. Amplifications were performed in 25 μL reactions, each containing 2.5 μL of a non-diluted DNA template, 12.5 μL of a KAPA HiFi HotStart Ready Mix containing KAPA HiFi HotStart DNA Polymerase, a buffer, MgCl_2_, dNTPs (KAPA Biosystems Inc., Wilmington, MA, USA) and 5 μL of each primer at 1 μM. Thermal cycling conditions consisted of an initial denaturation step (3 min at 95 °C), followed by 30 cycles of denaturation (30 s at 95 °C), annealing (30 s at 55 °C), and extension (30 s at 72 °C). These were followed by a final extension step of 10 min at 72 °C. Once the desired amplicon was confirmed in 1% agarose gel, electrophoresis followed by SYBR safe staining (ThermoFisher Scientific, Inc., Waltham, MA, USA) and PCR products were stored at −30 °C until sequencing library preparation. Amplified DNA templates were cleaned of non-DNA molecules as well as Illumina sequencing adapters (AMPure XP beads, Illumina, Inc., San Diego, CA, USA), and dual indices were attached using a Nextera XT Index Kit (Illumina, Inc.) and the corresponding PCR amplification program, as described in the MiSeq rRNA Amplicon Sequencing protocol (Illumina Inc.). After the second round of clean-up (AMPure XP beads, Illumina, Inc.), amplicons were quantified using a Qubit dsDNA BR assay (ThermoFisher Scientific, Inc.) and diluted to equimolar concentrations (4 nM) for further pooling. Sequencing was performed on an Illumina MiSeqTM platform (Illumina Inc.) at the IGTP, according to the manufacturer’s specifications, to generate paired-end reads of 300 base-length in each direction (Accession numbers PRJEB52963 and PRJEB52964).

### 2.4. 16S rRNA Sequence Analysis

The quality of raw sequencing data was visualized using FastQC (v0.11.9) [74] and DADA2 software (v1.10.1) [75]. The pipeline was executed according to default parameters using maxEE = 4.10 in the filtering step. After removing chimeric reads, amplicon sequence variant (ASV) tables were generated, and high-quality reads were classified against the Ribosomal Database Project (RDP) [76]. Downstream analyses were conducted in R (v3.5.2) [77] using phyloseq (v1.26.1) [78], vegan (v2.5.5) [79] ade4 (v1.7.13) [80] and ggplot2 (v3.2.0) [81] packages. Alpha diversity (Shannon and Chao1 indexes) was estimated using the R/phyloseq ‘estimate_richness’ function. Beta diversity based on Bray–Curtis distances were visualized using principal coordinate analysis (PCoA), and differences were evaluated using a PERMANOVA (adonis) test. Differences among groups were evaluated using non-parametric Wilcoxon signed-rank and Kruskal–Wallis tests. An unadjusted *p*-value ≤ 0.05 was considered of statistical significance unless otherwise specified. Associations between cytokines and bacteria were determined based on Spearman correlation coefficients using the rcorr function within the R/hmisc package (v4.6.0).

### 2.5. Overlapping Peptides Covering the HTI Sequence

As a recall antigen for immunological studies, a set of 147 overlapping peptides (OLP) of 15 amino acids in length, overlapping by 11 amino acids and spanning the complete HTI sequence, were designed using the PeptGen Peptide Generator [82] and the Los Alamos HIV database and synthesized (Synepeptide, Shanghai, China). To measure T-cell responses to HTI, 147 OLPs were grouped into 17 peptide pools according to the HIV protein subunit fragments they covered (Supplementary Table S1): 7 for Gag (11–8 peptides/pool), 7 for Pol (11–5 peptides/pool), 2 for Vif (8 and 7 peptides/pool) and 1 for Nef (2 peptides/pool).

### 2.6. Mouse IFNγ ELISPOT Assay

IFNγ ELISPOT assays were performed using mouse IFNγ ELISPOT ALP reagents (Mabtech AB, Nacka Strand, Sweden) following the manufacturer’s instructions with minor modifications. Briefly, cryopreserved spleen cells from mice in the mock (n = 16, 8 females and 8 males), DDDCM (n = 16, 7 females and 9 males), and DDDCM-ATB (n = 13, 6 females and 7 males) groups were thawed, washed, and counted (NucleoCounter^®^ NC-3000, Chemometec, Allerod, Denmark). For the six independent validation groups tested using fresh cells, isolated spleen cells were washed and counted. Spleen cell density was adjusted with R10, and 4 × 10^5^ cells/well were plated in 96-well polyvinylidene plates (Millipore Corp., Burlington, MA, USA), coated with an IFNγ capture antibody (clone AN-18). Subsequently, cells were stimulated with the 17 HTI-specific OLP pools mentioned above (Table S1) at a final concentration of 14 μg/mL for each peptide for 16 h at 37 °C in 5% CO_2_. Concanavalin A (Sigma-Aldrich Corp) at 5 µg/mL was used as a positive control, and R10 in triplicate as a negative control. After stimulation, spot-forming cells (SFC) were revealed by adding a biotinylated IFNγ detection antibody (clone R4-6A2), streptavidin conjugated with alkaline phosphatase (AP), and the AP Conjugate Substrate Kit (Bio-Rad Laboratories, Hercules, CA, USA) consecutively. SFCs per well were counted using an automated ELISPOT reader system (Cellular Technology Limited Analyzers LLC, Cleveland, Ohio, USA), together with ImmunoSpot software (v7.0.21.3), and the results were adjusted to SFC/10^6^ spleen cells. The threshold for positive responses was defined for each animal as responses exceeding all the following criteria: (i) a minimum of 50 SFC/10^6^ spleen cells, (ii) mean SFC/10^6^ spleen cells in negative control wells plus three standard deviations of the negative control wells, (iii) three times the mean SFC/10^6^ spleen cells of negative control wells. Animals with thresholds higher than 200 SFC/10^6^ spleen cells were considered overactivated and corresponding data were excluded. Pools showing responses below the threshold were considered non-responding pools and not summed in the total magnitude. Results are reported as the total magnitude (the sum of responses above the threshold to all peptide pools) and breadth (the number of positive peptide pools).

### 2.7. Luminex Assay

The serum concentration of CD40 Ligand (CD154), GM-CSF, IFNγ, IL-1ß, IL-2, IL-4, IL-5, IL-6, IL-10, IL-12 (p70), IL-13, IL-15, IL-17A, IL-17E/IL-25, IL-21, IL-22, IL-23, IL-27, IL-31, IL-33, TNFα and TNFß were measured in samples from the mock (n = 10, 5 females and 5 males), DDDCM (n = 17, 8 females and 9 males) and DDDCM-ATB (n = 13, 6 females and 7 males) groups at the final study time point (week 21). Briefly, frozen serum samples were thawed, vortexed, and centrifuged at 10,000× *g* for 10 min before 25 µL of the serum was collected for further testing. The serum concentration of 22 cytokines was measured simultaneously using duplicate samples in a customized mouse Th17 magnetic bead panel kit (Milliplex, Millipore Corp.) and a Luminex^®^ 200 reader (Luminex Corp, Austin, TX, USA), following the manufacturer’s instructions.

### 2.8. Statistical Analysis of Vaccine Response Data

Results were analyzed with GraphPad Prism version 9 software using non-parametric tests. Differences between medians were evaluated using the Mann–Whitney U test. When multiple tests were performed, the Kruskal–Wallis test with a false discovery rate (FDR) for multiple comparisons was applied. Statistical significance was set at two-sided *p* < 0.05 and q < 0.05 when necessary.

## 3. Results

### 3.1. Vaccination with a Highly Immunogenic Prime-Boost Strategy Triggers Strong HTI-Specific IFNγ Producing T-Cell Responses despite Depleted Microbiota

We tested vaccine immunogenicity in C57BL/6 mice using three DNA.HTI (DDD) prime immunizations followed by a boost with one dose of ChAdOx1.HTI and one further dose of MVA.HTI (CM). Mice with intact (DDDCM) and depleted (DDDCM-ATB) microbiota were compared for HTI-specific T-cell responses. The results obtained in these groups were compared with a placebo arm (mock) with an unmodified gut microbiome (Figure 1).

Before animal vaccination, we performed a fecal microbiota transplant (FMT) to homogenize the microbiota between groups. To evaluate the extent of FMT engraftment, microbial composition and diversity analyses were conducted (Supplementary Data S1). The gut microbiota composition of mice in the mock and DDDCM groups was homogeneous after FMT and comparable with the microbiota composition at the time of animal arrival (Figure S1). We also assessed, via the longitudinal 16S rRNA gene sequence profiling, the extent of gut microbiota depletion due to sustained antibiotic treatment in the DDDCM-ATB group (Supplementary Data S2). As expected, the gut microbiota was largely depleted. Some 16S rRNA amplifications of DDDCM-ATB samples failed, indicating a low bacterial biomass. In the sequenced samples, both the number of reads (>50% reduction compared to mock and DDDCM) and bacterial diversity were largely reduced in DDDCM-ATB samples (Figure S2) and clustered separately from samples obtained from mock and DDDCM animals. Remarkably, we detected murine mitochondrial 16S RNA sequences in the DDDCM-ATB group (with a global mean abundance of 32%), showing that a proportion of the amplified DNA was derived from non-bacterial sources. We also observed that male mice in the DDDCM-ATB group gained less weight than males with normal microbiota (Figure S3). In addition, we reported the death of four mice in the DDDCM-ATB group without any previous clinical signs.

**Figure 2:**
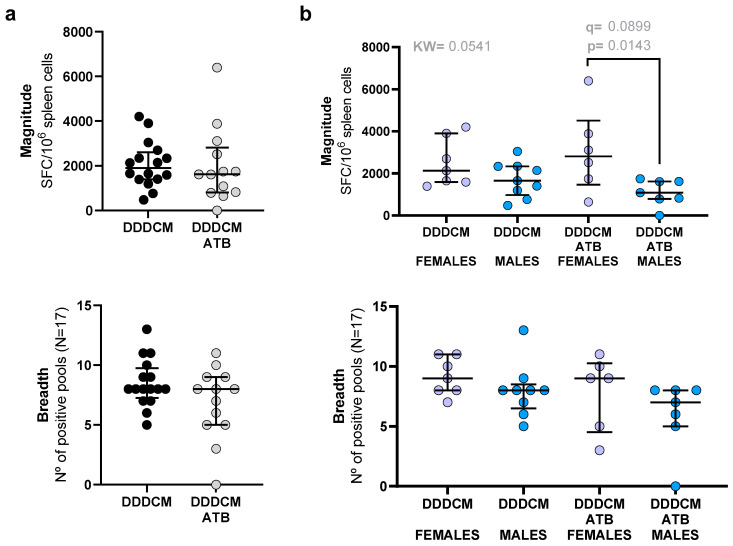
Magnitude and breadth of IFNγ-producing, HTI-specific T-cell responses after vaccination without (DDDCM) and with (DDDCM-ATB) microbiota depletion. Median and interquartile ranges of the magnitude and breadth are indicated. Data are shown by groups (**a**) and segregated by sex (**b**). DDDCM (n = 16, 8 females and 8 males) and DDDCM-ATB (n = 13, 6 females and 7 males) groups were compared. The Mann–Whitney U test and Kruskal–Wallis (KW) test with a false discovery rate (FDR) for multiple comparisons were used to compare groups. Statistically significant differences were set at *p* < 0.05 and q < 0.05 when FDR was used. Trends (*p* < 0.05 and q < 0.1) are indicated in grey.

Since HTI immunogen was designed based on IFNγ T-cell responses, levels of IFNγ-producing spleen cells were used as the main read-out for vaccine-induced T-cell immunity. Three weeks after the last immunization, the unspecific activation of T-cells, measured as IFNγ-producing unstimulated spleen cells (mean of the negative triplicate wells per animal), was slightly higher in the DDDCM group compared to the mock vaccinated group (median mock: 4 SFC/10^6^ spleen cells vs. DDDCM: 8 SFC/10^6^ spleen cells, *p* = 0.0216, q = 0.0680), suggesting that vaccination increased basal global T-cell activation (Figure S4). This difference was not detected in the DDDCM-ATB group, which showed lower basal activation levels than the DDDCM group (median DDDCM-ATB: 5 SFC/10^6^). After specific re-stimulation with 17 overlapping peptides (OLP) pools covering the HTI sequence (Table S1), spleen cells derived from mock-vaccinated mice showed no antigen-specific IFNγ responses above the threshold while, as expected, vaccinated animals showed strong IFNγ secreting T-cell responses in terms of magnitude (median mock = 0, DDDCM = 1894, DDDCM-ATB = 1616 SFC/10^6^ spleen cells) and breadth (median mock 0, DDDCM = 8, and DDDCM-ATB = 8 positive peptide pools) (Figure 2).

Our initial analysis indicated that depleting the microbiota had no effect on the response to the DDDCM heterologous prime-boost regime with HTI in terms of the magnitude and breadth of IFNγ-producing T-cells (Figure 2a). However, when stratifying by sex, we observed that, in the DDDCM-ATB group, females showed responses of higher total magnitude compared to males (median DDDCM = 2812 SFC/10^6^ spleen cells vs. DDDCM-ATB = 1085 SFC/10^6^ spleen cells, *p* = 0.0143, q = 0.0899; Figure 2b). These differences did not translate into significant differences in the breadth of the response between males and females.

We used data from additional independent experiments to further investigate the potential sex-specific effect of the microbiota depletion. We compared IFNγ T-cell responses to HTI between males and females after vaccination with the following: (i) DDD, (ii) DDDM, (iii) C, (iv) CM, and (v) DDDCM. Groups (i) to (v) had intact microbiota. A sixth group, with ATB-depleted microbiota, was vaccinated with DDDCM. In this additional microbiota-depleted group (DDDCM-ATB2), we also observed a trend toward lower responses in males compared to females. However, we observed a similar trend in the CM-vaccinated group with an intact microbiota (Figure S5). Thus, differences in vaccine response between females and males were not exclusively seen in microbiota-depleted mice and could also be related to the vaccination regimen.

### 3.2. Gut Microbiota and DDDCM.HTI Vaccination Affect Serum Cytokine Levels

**Figure 3:**
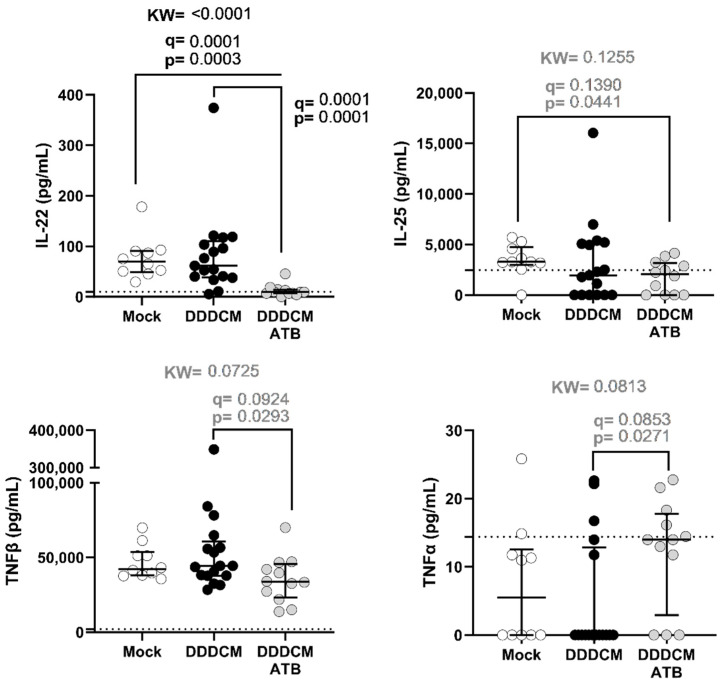
Serum levels of cytokines showing at least one significant difference between study groups (*p* < 0.05 in the Kruskal–Wallis (KW) test with an FDR correction for multiple comparisons) when comparing mock (n = 10)-vaccinated mice (DDDCM, n = 17) and mice vaccinated with depleted microbiota (DDDCM-ATB, n = 12). The lower limit of quantification is indicated by a dotted line, and median and interquartile ranges are shown. The statistically significant threshold was set at *p* < 0.05 and q < 0.05, and trends (*p* < 0.05, q > 0.05) are indicated in grey.

Since IFNγ T-cell responses to the HTI vaccine in spleen cells were not severely affected by microbiota depletion, we decided to test 22 soluble markers that could be produced by intestinal cells after microbiota depletion that could impact the T-cell response to intramuscular vaccination. Of the 22 cytokines tested in the serum, taken at the last study time point three weeks after the last vaccination, we detected statistically significant differences (*p* < 0.05) between groups for IL-22, IL-25 (or IL-17E), TNFα, and TNFβ (Figure 3). However, only IL-22 remained significantly altered after correcting for the false discovery rate (mock vs. DDDCM *p* = 0.0003, q = 0.0001 and mock vs. DDDCM-ATB *p* = 0.0001, q = 0.0001). In addition, TNFβ serum levels were reduced in animals with depleted microbiota (DDDCM vs. DDDCM-ATB *p* = 0.0293, q = 0.0924), while IL-25 was reduced in vaccinated animals with depleted microbiota compared to non-vaccinated animals (mock vs. DDDCM-ATB *p* = 0.0441, q = 0.1390). Finally, we identified TNFα to be increased in microbiota-depleted and vaccinated animals (DDDCM vs. DDDCM-ATB *p* = 0.0271, q = 0.0853).

We also compared the serum levels of these four cytokines when segregating animals by sex to identify potential sex-related differences in microbiota-depleted animals (Figure S6). In the DDDCM-ATB group, none of these four cytokines showed differences in serum levels between males and females. However, the differences in IL-22 serum levels between the mock and the DDDCM-ATB group were maintained in females and males separately (females *p* = 0.0126, q = 0.0264 and males *p* = 0.0105, q = 0.0105). IL-22 serum levels were also significantly higher in males from the DDDCM group compared to males in the DDDCM-ATB group (*p* < 0.0001, q = 0.0008). In addition, we observed that the lower TNFβ levels in serum observed in animals with depleted gut microbiota were mainly caused by decreased levels in females. On the other hand, IL-25 differences showed a trend of lower levels in males after vaccination (for the DDDCM and DDDCM-ATB groups) compared to mock-vaccinated males. The differences observed for TNFα were not maintained when males and females were analyzed separately.

### 3.3. IL-22 Serum Levels Correlate with the Abundance of Short Chain Fatty Acid (SCFA) Producing Bacteria in Large Intestine

**Figure 4:**
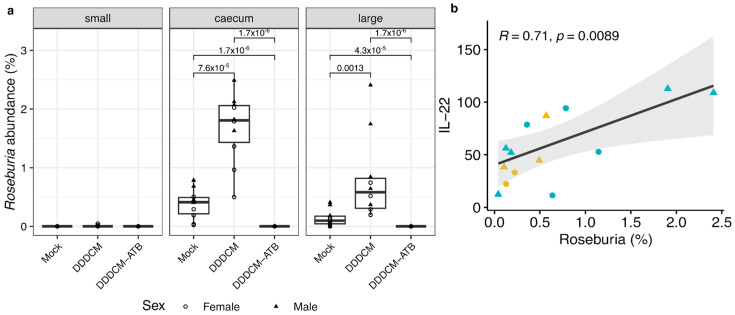
(**a**) Relative abundance of *Roseburia* in the caecum, large, and small intestine sections for the three different groups: the mock (n = 10 per sample type), vaccinated DDDCM (n = 10 per sample type), and vaccinated with depleted microbiota DDDCM-ATB (n = 16 per sample type) groups. Comparisons were performed using the Kruskal–Wallis test, boxplots, and statistically significant differences were indicated (*p* < 0.05). (**b**) Spearman’s correlation between IL-22 levels in serum and *Roseburia* abundance in the large intestine of DDDCM (n = 9) and mock (n = 5) groups. Correlation coefficient and *p*-value are indicated.

Next, we sought to explore if the observed reduction in IL-22 production after microbiota depletion could be influenced by bacteria species producing specific metabolites, as suggested by previous evidence [24,83,84]. To this end, we tested if the abundance of 19 SCFA-producing bacteria [85] in the small, caecum, and large intestine correlated with IL-22 levels in the serum (Supplementary Table S2). A strong correlation (R = 0.71, *p* = 0.0089) between the abundance of *Roseburia* in the large intestine and the level of IL-22 in the serum was observed. The abundance of this bacteria was higher in DDDCM-vaccinated compared to mock-vaccinated mice, but it was not detected in vaccinated animals with depleted microbiota (Figure 4).

## 4. Discussion

Only a few studies have addressed to what extent the gut microbiota could influence the T-cell’s immune response to vaccination. In this study, we compared HTI-specific T-cell responses between mice with intact and ATB-depleted microbiota. HTI was chosen as the model antigen as it has been shown to be highly immunogenic in C57BL/6 and BALBc mice [62,64,86] as well as in human clinical trials [69] using the same read-out, IFNγ ELISPOT. This allowed us to test the impact of the gut microbiota on HTI-specific T-cell vaccine responses using a sensitive and high throughout-put methodology and translate the results to the human setting. This study was carried out in specific pathogen-free mice instead of gnotobiotic mice to allow the normal development of the immune system, which requires the presence of intestinal microbiota [3]. Once mice had developed their immune system, the gut microbiota was depleted in one group to generate secondary abiotic mice [87,88] using a combination of four wide-spectrum antibiotics.

To reduce potential basal variability in microbiota composition, a mouse-to-mouse FMT was performed before starting the experiments. We observed that the gut microbiota composition after FMT was not significantly different between the mock and DDDCM groups. However, the high microbiota similarity between groups before FMT, along with the low bacterial viability of the FMT donor sample, made it difficult to confirm that FMT was fully grafted. It is also possible that the intestinal tract was repopulated with bacteria that remained in the animal’s intestine after ATB treatment. These data also suggest that animals sharing the same origin over a short period of time already have very similar microbiota. Consequently, FMT could be substituted by less invasive treatments to homogenize the microbiota, like mixing shavings, thereby increasing overall animal welfare.

We used a gut microbiota-depleted group (DDDCM-ATB) to study the impact of the microbiota on the response to HTI vaccination. Microbiota depletion was confirmed in longitudinal feces samples and the intestinal content via 16S rRNA gene sequencing. Samples from the DDDCM-ATB group showed a one-half reduction in read counts and an elevated presence of mitochondrial sequences, probably from desquamated murine intestinal cells. Altogether, the results indicate that after the ATB treatment, the microbiota was significantly reduced, if not almost eliminated.

Depleting the microbiota had a limited effect on the T-cell response to vaccination, at least when measured as IFNγ-producing spleen cells in C57BL/6 mice. However, we cannot rule out the fact that in mice with a different microbiota composition, due to different genetic backgrounds, diet, or origin, microbiota depletion could have a different effect on immune responses. Although the IFNγ ELISPOT read-out employed cannot differentiate between cell types, after cryopreservation, using 15 mer peptide stimulations and an 18 h incubation time, the IFNγ responses measured are likely to be mostly CD8+ T-cell mediated [89,90]. Since the vaccination regimen used in the present study was highly immunogenic, this could have reduced the impact of microbiota depletion on the response to vaccination. This is in line with previous studies in mice, showing that the immunogenicity of adjuvanted vaccines is not affected by the microbiota, while nonadjuvanted vaccines profit significantly from the presence of the microbiota, probably due to TLR5 stimulation via bacterial flagellin [2,34]. We did not use adjuvanted vaccines in our study, but the two viral vectors used were large viruses containing different PAMPs, which can have similar immunostimulatory effects as adjuvants. It is possible that less immunogenic vaccine vectors are more affected by the microbiota, a question that deserves further investigation. For instance, nucleic acid (DNA and RNA)-vectored vaccines lacking PAMP expression could be more susceptible to microbiota changes and might need to be adjuvanted to avoid it.

We observed reduced responses in males from the vaccinated, antibiotic-treated group compared to females. However, this was also observed in independent studies with vaccinated animals without depleting the microbiota and could not be attributed exclusively to a lack of microbiota. Indeed, males and females from the DDDCM-ATB group were clustered together in PCoA analysis (Figure S2), indicating a similarity in gut microbiota composition. In addition, the low number of animals probably limited the capacity to observe sex-related differences in other groups. Indeed, previous reports have shown that male animals commonly respond weaker than females [91,92]. Nevertheless, these results highlight the need to use both sexes in animal experiments to capture potential sex-related differences.

We also explored whether the depletion of the gut microbiota could cause changes in the immune response beyond IFNγ T-cell responses. Consequently, we measured serum levels of 22 anti- and pro-inflammatory cytokines that could be produced by immune cells in the intestinal mucosa and regulate T-cell responses to vaccination in lymphoid tissues. We found that IL-22 was significantly reduced in the serum of mice with depleted microbiota. Interestingly, gut microbiota indirectly promotes the production of IL-22 by CD4+ T-cells and innate lymphoid cells (ILC). Several microbial species have been described to produce short-chain fatty acids (SCFA), which are detected by the G-protein receptor 41 and 43 (GPR41 and GPR43) on CD4+ T-cells and ILC, promoting the expression of IL-22 [24,83,84]. On the other hand, it has also been reported that a decrease in propionate production by reducing the gut microbiota with vancomycin treatment resulted in a decrease in IL-17 and IL-22 production by γδ T-cells [93]. Remarkably, we also included vancomycin in our antibiotic treatment to deplete the microbiota. Thus, it is plausible that a loss of SFCA production after microbiome depletion caused a strong reduction in IL-22 production via ILC and T-helper cells in the gut. We tested this hypothesis by correlating the abundance of SCFA producers and serum IL-22 levels. Our data show that IL-22 serum levels were positively correlated with the presence of *Roseburia*, butyrate and propionate-producing bacteria, in the large intestine of animals with intact microbiota (mock and DDDCM). This correlation was further supported by type 2 diabetes studies in mice where the relative abundance of *Roseburia intestinalis* was associated with higher IL-22 production [94]. In our experiments, the abundance of *Roseburia* increased after DDDCM vaccination compared to the mock group, while in the DDDCM-ATB group, *Roseburia* was not detected. The mechanisms by which DDDCM vaccination could increase the presence of this bacteria are not clear and deserve further study. Of note, the anti-inflammatory properties of *Roseburia* could represent an advantage when adapting to local intestinal inflammation caused by soluble inflammation mediators produced after intermuscular vaccination. In addition, it is possible that this increasing abundance of *Roseburia* could counteract the adverse effects of soluble mediators of inflammation in the intestine [95,96]. Together, these results further support the important role of SCFA-producing bacteria, such as *Roseburia*, in regulating the production of IL-22 by mucosal immune cells.

The effects of IL-22 on T-cell responses have been poorly described and are supposed to be indirect due to the lack of expression of the IL-22 receptor on immune cells [97]. IL-22 is mainly an inflammatory mediator produced by innate (ILC, neutrophils, and macrophages) and adaptive T-cells (Th1, Th17, and Th22). It has been shown to up-regulate innate immune responses, acting as a homeostatic factor for tissue regeneration, and has also been linked to different inflammatory diseases driven by T-cells [98]. The production of IL-22 by Th17 cells and ILC in response to the intestinal microbiota has also been found to be essential to ensure the maintenance of intestinal barrier function [83]. However, there are reports on the indirect and direct effects of IL-22 on T-cell polarization in different disease contexts, some of which relate IL-22 signaling with reduced Th1 responses. It is thus possible that a reduction in IL-22 could result in an increase in Th1-polarized IFNγ-producing T-cells, although this was not observed in our experiments.

In addition to IL-22, the lower TNFβ serum levels detected in animals with depleted gut microbiota could be indicative of alterations in the gastrointestinal epithelium and the structure of Peyer Patches [99]. We also observed that the DDDCM.HTI vaccination reduced serum levels of IL-25, especially in male mice. This cytokine is known to support Th2 responses by inducing the production of IL-4, IL-5, and IL-13 and reducing IFNγ production. It is, thus, possible that this reduction promotes IFNγ and Th1 responses, which are the main targets of T-cell-based therapeutic vaccinations to promote the control of HIV infection.

## 5. Conclusions

The results presented here show that, in the mouse strain used and with the heterologous prime-boost vaccination regimen tested, the antibiotic-mediated depletion of murine gut microbiota results in a profound decrease in serum levels of IL-22, especially related to the loss of SCFA-producing *Roseburia* bacteria. However, this was not reflected by changes in the magnitude of IFNγ T-cell responses after HTI vaccination. Therefore, our data show that even if the capacity of the immune system to respond to vaccination is reduced due to a profound perturbation of microbiota, poor T-cell reactogenicity may be overcome by a highly immunogenic vaccination regimen. This is especially relevant when considering the effect of the gut microbiota on immune responses and its vulnerability to external factors such as age, diet, drug exposure, or disease [2,16,32]. However, the changes in cytokine levels detected in this study, especially IL-22, suggest that alternative responses aside from IFNγ-mediated Th1 polarization, such as Th17, Th22, and mucosal responses, could be affected and need to be further studied.

## Data Availability

The raw sequence data presented in this study are openly available in the European Nucleotide Archive (ENA) under accession numbers PRJEB52963 and PRJEB52964.

## Supplementary Materials

The following supporting information can be downloaded at: https://www.mdpi.com/article/10.3390/vaccines11111663/s1, Supplementary Data S1: Fecal microbiota transfer (FMT) engraftment; Figure S1: PCoA and bacterial diversity analysis before and after FMT; Supplementary Data S2: Antibiotic-driven microbiota depletion in mice receiving HTI vaccination; Figure S2: Microbiota composition changes after bacterial depletion with ATB treatment; Figure S3: Mice weight before euthanasia (week 20); Figure S4: Basal spleen cell activation; Figure S5: Comparison between males and females of the IFNγ response to different vaccination regimes with unmodified microbiota (DDD, DDDM, C, CM, DDDCM) or with an antibiotic-depleted microbiota (DDDCM-ATB2); Figure S6: Differences in cytokine levels segregated by sex; Table S1: Overlapping peptide pools for IFN γ ELISPOT testing; Table S2: Correlogram of the relative abundance of short-chain fatty acid (SCFA) producers with IL-22 serum levels.
