# Supplementary material for: Vaccination with an HIV T-Cell Immunogen (HTI) Using DNA Primes Followed by a ChAdOx1-MVA Boost Is Immunogenic in Gut Microbiota-Depleted Mice despite Low IL-22 Serum Levels
Source: Vaccines (Basel). 2023 Oct 30;11(11):1663. doi: 10.3390/vaccines11111663 (PMC10675013; doi:10.3390/vaccines11111663)
Supplement: Supplementary file 1 [file vaccines-11-01663-s001.zip › vaccines-2622967-supplementary.pdf]

Supplementary data

Supplementary Data S1. Fecal microbiota transfer (FMT) engraftment.

Before animal vaccination, we performed a fecal microbiota transfer (FMT) to homogenize the microbiota among groups. Microbial composition and diversity analyses were performed to evaluate the extent of FMT engraftment. We observed high between-groups baseline similarity, with samples at week 0 from all groups clustering together (Fig. S1a). As expected, antibiotic (ATB) conditioning before FMT (week 1) reduced the number of reads and the bacterial diversity (Shannon index p-value = 0.00016) in all experimental groups (Fig. S1b). This was further supported by the PCoA analysis since all samples at week 1 clustered together and separated from the other time points in the three groups. Bacterial diversity in FMT donors and feces from the three experimental groups sampled at animal arrival (week 0) was similar. Of note, an assessment of FMT donor samples viability after storage at -80°C showed that the bacterial viability was remarkably low (3.8%). After FMT, fecal samples (week 2) did not show significant differences in bacterial diversity (Shannon index p-value=0.85) compared to samples obtained at animal arrival (week 0); whereas a significant decrease in diversity (Shannon index p-value = 0.031) was observed after ATB conditioning for FMT (week 1). Accordingly, beta diversity analysis (Fig. S1a) showed that samples from week 0 and week 2 were comparable and clustered together with FMT-donor samples, except for samples from the DDDCM-ATB group. Altogether, these results indicate that bacterial diversity was recovered one week after FMT (Fig. S1), except in the DDDCM-ATB group that started ATB treatment in drinking water at the time that FMT was performed (week 0). These results indicate that the gut microbiota composition of animals in the mock and DDDCM groups were homogeneous after FMT and comparable with the microbiota composition at animal arrival.

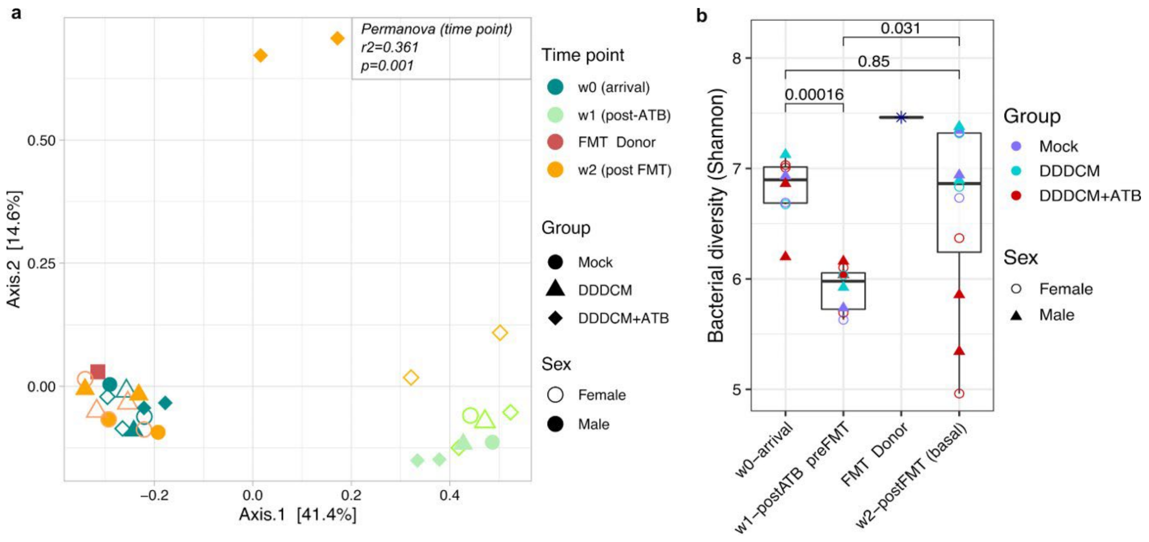

***Supplementary Figure S1**. PCoA and bacterial diversity analysis before and after FMT (a) PCoA based on Bray-Curtis distances (ASV-level). PERMANOVA r-squared and p-values are shown. (b) Boxplots representing Shannon alpha diversity (ASV-level). P-values based on the Kruskall-Wallis test are shown. Comparisons were performed between FMT donor samples and the three experimental groups (Mock-vaccinated, DDDCM-vaccinated, and vaccinated with DDDCM while depleting the microbiota with ATB) at week 0 (arrival), week 1 (after ATB treatment for FMT), and week 2 (post-FMT).*

Supplementary Data S2. Antibiotic-driven microbiota depletion in mice receiving HTI vaccination.

16S rRNA gene-based profiles were assessed to confirm gut microbiota depletion in the DDDCM-ATB group. The ATB treatment proved to be effective, as three longitudinal fecal samples from the DDDCM-ATB group (weeks 2, 11, and 17) showed no 16S rRNA gene region amplification by agarose gel electrophoresis and SYBR Safe staining. In addition, 16S rRNA PCR amplification failed in 3 out of 12 small intestine samples and 6 out of 12 caecum samples, providing initial evidence that the bacterial microbiota was largely depleted in the DDDCM-ATB group samples.

After sequencing, a mean of approximately 161,000, 153,000, and 76,000 raw 16S rRNA gene reads (paired-end) were generated for longitudinal Mock, DDDCM, and DDDCM-ATB group samples. Both extraction and non-template negative control had less than 15,000 filtered reads (mean reads: 1,608 extraction and 12,153 non-template PCR). Inter-group comparisons showed no significant differences in high-quality filtered bacterial reads between Mock and DDDCM groups, whereas both groups showed significantly higher sequencing yield when compared to the DDDCM-ATB group (Fig. S2a). No significant differences were observed between Mock and DDDCM over time, whereas both Mock and DDDCM groups showed significantly higher alpha diversity compared to the DDDCM-ATB group, from week 2 until the end of the study (Fig. S2b).

Moreover, PCoA analysis based on Bray-Curtis distances showed that Mock and DDDCM samples clustered together and separately from DDDCM-ATB samples, at all time points after FMT (week 2) as well as at the last time point of the study (Fig. S2c).

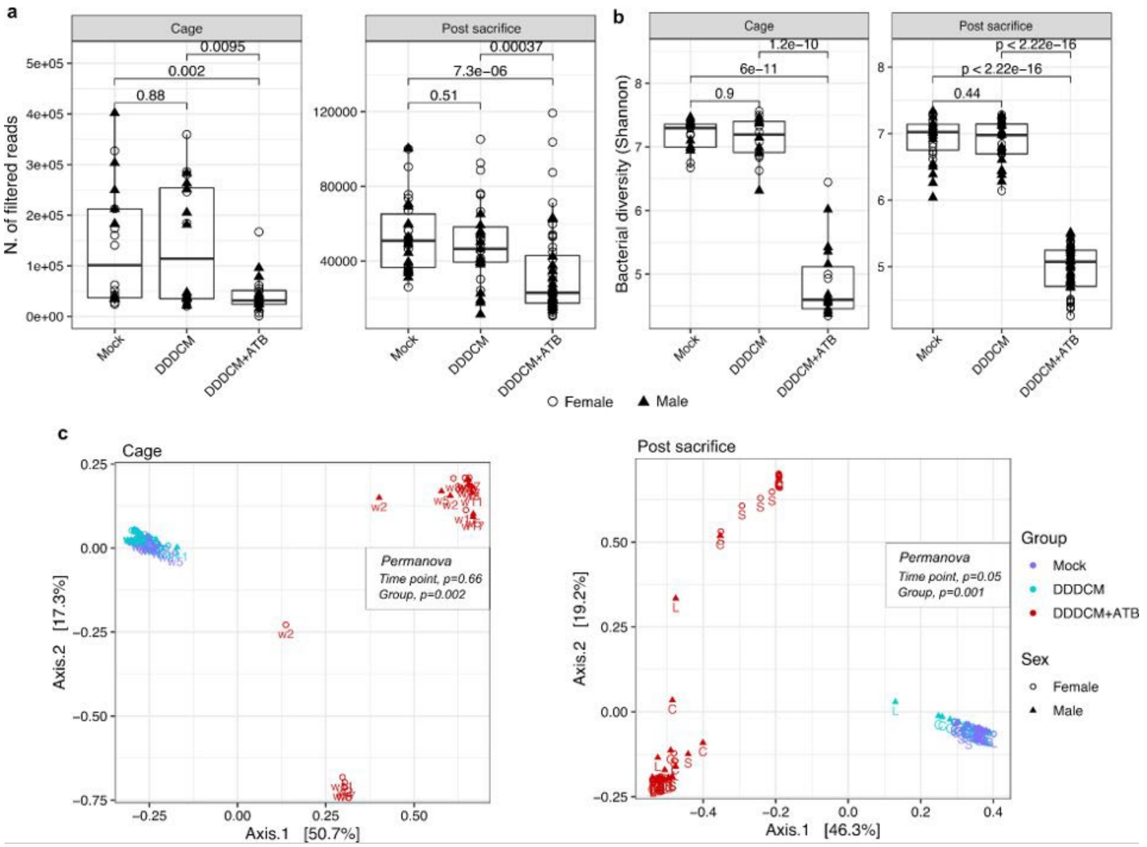

Supplementary Figure S2. *Microbiota composition changes after bacterial depletion with ATB treatment. (a) Bacterial reads and (b) Shannon diversity in the DDDCM-ATB group were compared using box plots and the Kruskall-Wallis test to Mock (PBS) and DDDCM groups at the different study time points after FMT (weeks 2 to 18) as well as in the small (S), cecum (C) and large (L) intestine at the last study time points. (c) PCoA analysis during the different study time points after FMT (weeks 2 to 18) and in the small, cecum, and large intestines at the study's last time point. PERMANOVA r-squared and p-values are shown.*

The PCoA analysis revealed that the bacterial composition was markedly different in group DDDCM-ATB compared to Mock and DDDCM groups throughout the experiment (Fig. S2c). In addition, we detected murine mitochondrial 16S RNA sequences in the DDDCM-ATB group (global mean abundance of 32%), showing that a portion of the amplified DNA was derived from a non-bacterial source. Also, some bacterial sequences detected in the low-biomass DDDCM-ATB samples were found in negative DNA extraction and PCR controls (data not shown). Thus, the ATB treatment massively reduced the bacterial gut microbiota in group DDDCM-ATB.

Finally, we observed that male mice with ATB-depleted microbiota gained less weight than males with an untreated microbiota, no matter if they were Mock or DDDCM vaccinated (p = 0.0009 and 0.0067, q < 0.05 at the last study time point; Fig. S3). Of note, this effect was not observed in female mice. In addition, four animals in the DDDCM-ATB group (2 males and 2 females) died before finishing the experiment, without any previous clinical signs. Necropsy and anatomopathological studies of the gut indicated that the animals had cecum distension and hemorrhage, which would have probably caused a segmental torsion of the cecum.

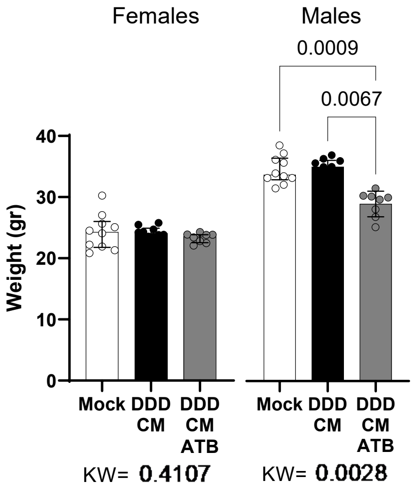

Supplementary Figure S3. *Mice weight before euthanasia (week 20). Weights were compared between groups in males and females, bars represent median values, and interquartile ranges are also shown. Statistical significative differences were evaluated using the Kruskal-Wallis test (KW) with false discovery rate (FDR) correction for multiple comparisons. Significative statistical differences (p < 0.05, q < 0.05) between groups are indicated in the graph.*

Supplementary figures.

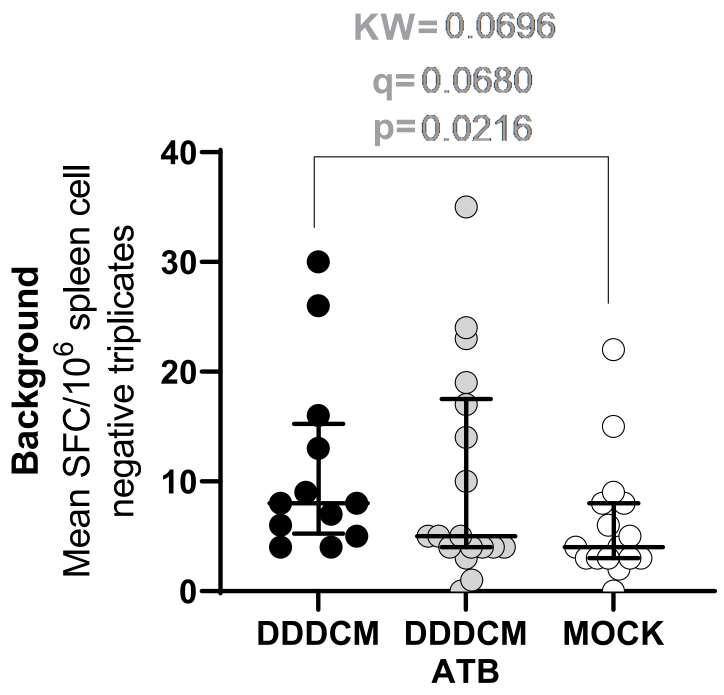

Supplementary Figure S4. *Basal spleen cell activation. The mean and standard deviation of the three negative controls per animal are indicated. Differences between groups were evaluated using the Kruskal-Wallis test (KW) with FDR correction for multiple comparisons. Trends ($p < 0.05$, $q < 0.1$) between group results are indicated in the graph in grey.*

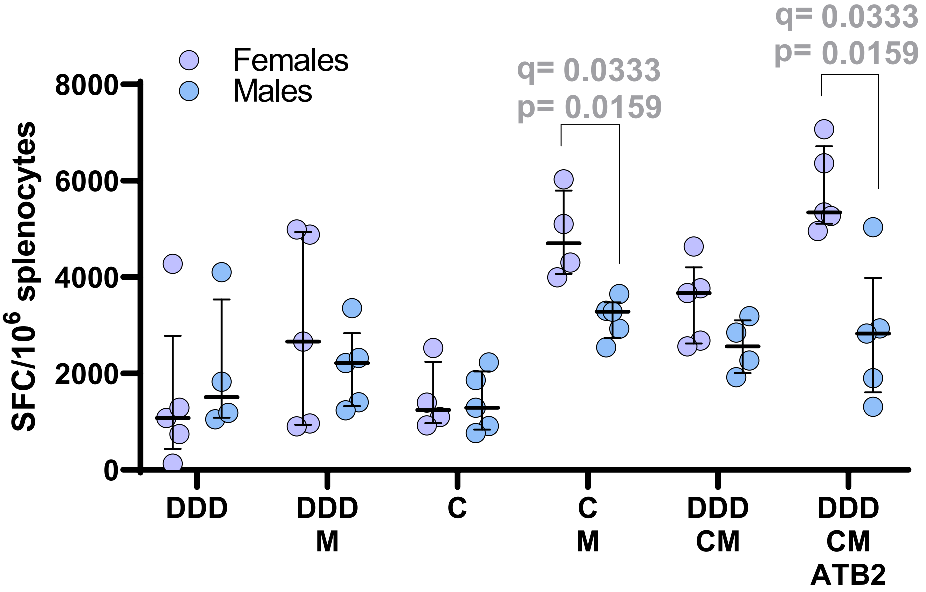

Supplementary Figure S5. *Comparison between males and females of the IFNγ response to different vaccination regimes with unmodified microbiota (DDD, DDDM, C, CM, DDDCM) or with an antibiotic-depleted microbiota (DDDCM-ATB2). Spot-forming cells (SFC) per million spleen cells in an IFNγ ELISPOT are indicated. The median and interquartile range are shown. Differences between males and females in the different groups were evaluated using multiple Mann-Whitney U tests with FDR correction for multiple comparisons, statistically significant differences were set at p < 0.05 and q < 0.05. Trends (p < 0.05 and q < 0.1) are indicated in grey.*

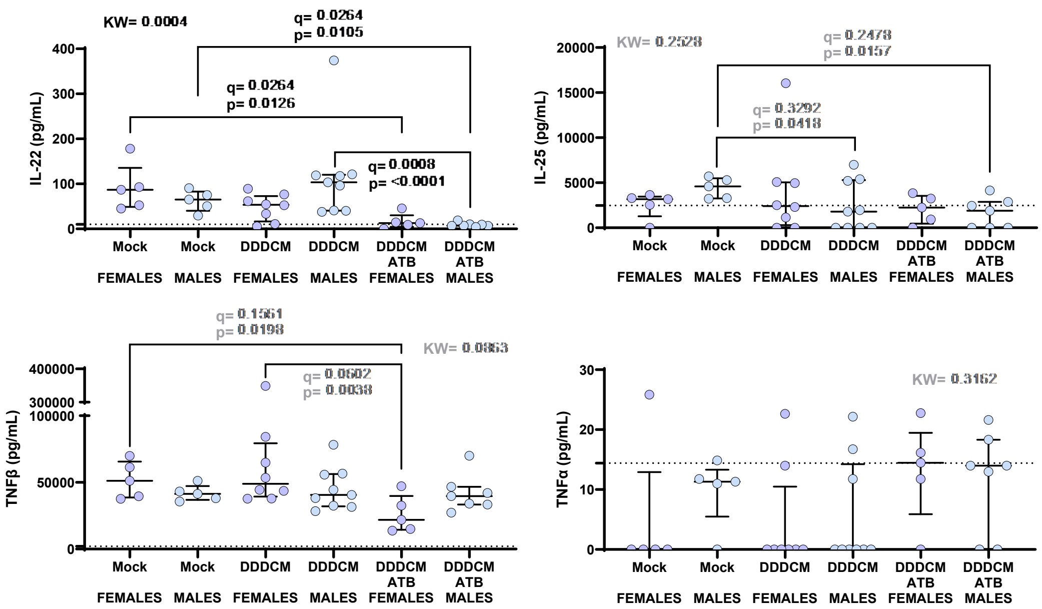

Supplementary Figure S6. *Differences in cytokine levels segregated by sex. Cytokine levels in mice serum showed at least one statistically significant difference in Kruskal-Wallis multiple comparisons (p < 0.05) with FDR correction for multiple comparisons after vaccination, in animals with or without depleting the microbiota, segregated by sex. Mock, vaccinated (DDDCM), and vaccinated with depleted microbiota (DDDCM-ATB) were compared, and median and interquartile ranges are shown. The lower limit of the quantification curve is indicated by a dotted line. The statistically significant threshold was set at p < 0.05 and q < 0.05, trends (p < 0.05, q > 0.05) are indicated in grey.*

Supplementary tables.

Supplementary Table S1. *Overlapping peptide pools for IFN γ ELISPOT testing*

ID	OLP per pool	HTI Segment	AA position in HXB2R protein	Source subunit	Source protein	% in HTI
1C	11	S1	17-50	p17	Gag	45%
1D	11	S1	51-94	p17		
1E	8	S2/3	162-170/193-203	p24		
1F	8	S4	223-245	p24		
1G	8	S4	246-282	p24		
1H	10	S5/6	296-308/350-363	p24		
1I	8	S7	426-452	acc		
1J	9	S8	101-121	PRT	Pol	44%
1K	8	S8	122-155	PRT		
1L	10	S9/10	189-205/365-372	RT		
2A	11	S10	373-419	RT		
2B	10	S11	464-496	RT		
2C	10	S12	925-958	Int		
2D	5	S13	981-996	Int		
2E	8	S14	26-50	-	Vif	8%
2F	7	S15	166-183	-		
2G	2	S16	57-68	-	Nef	3%

Supplementary Table S2. *Correlogram of the relative abundance of short-chain fatty acid (SCFA) producers with IL-22 serum levels. Genera excluded from correlation analysis due to 0 abundance across samples within each gut section are indicated by white squares. Statistically significant correlations are indicated by * p < 0.05, ** p < 0.01, *** p < 0.001.*

Genus	Small	Caecum	Large
Akkermansia			
Blautia			
Collinsella			
Coprococcus		-0,45	
Dialister			
Faecalibacterium			
Phascolarctobacterium			
Prevotella			
Subdoligranulum			
Alistipes	-0,059	-0,0066	0,024
Anaerostipes	-0,083	0,11	0,2
Bacteroides	-0,16	-0,36	-0,09
Bifidobacterium	0,34	0,13	0,28
Dorea		0,28	0,1
Roseburia	0,17	0,14	0,71 **
Ruminococcus		0,28	0,18
Eubacterium_oxidoreducens_group		0,42	0,22
Eubacterium_siraeum_group		-0,26	-0,2
Eubacterium_xylanophilum_group	0,5	0,11	0,29

Scale
1
0,8
0,6
0,4
0,2
0
-0,2
-0,4
-0,5
-0,6
-0,8
-1
